# Data mining of high density genomic variant data for prediction of Alzheimer's disease risk

**DOI:** 10.1186/1471-2350-13-7

**Published:** 2012-01-25

**Authors:** Natalia Briones, Valentin Dinu

**Affiliations:** 1 Computational Biosciences Program, School of Mathematics and Statistical Sciences, Arizona State University, 1711 South Rural Road, Tempe, Arizona, 85287-1804, USA; 2 Department of Biomedical Informatics, Arizona State University, Mayo Clinic, Samuel C. Johnson Research Bldg. 13212 East Shea Boulevard, Scottsdale, Arizona 85259, USA

**Keywords:** Late-Onset Alzheimer's Disease, GWAS, SNPs, Random Forest

## Abstract

**Background:**

The discovery of genetic associations is an important factor in the understanding of human illness to derive disease pathways. Identifying multiple interacting genetic mutations associated with disease remains challenging in studying the etiology of complex diseases. And although recently new single nucleotide polymorphisms (SNPs) at genes implicated in immune response, cholesterol/lipid metabolism, and cell membrane processes have been confirmed by genome-wide association studies (GWAS) to be associated with late-onset Alzheimer's disease (LOAD), a percentage of AD heritability continues to be unexplained. We try to find other genetic variants that may influence LOAD risk utilizing data mining methods.

**Methods:**

Two different approaches were devised to select SNPs associated with LOAD in a publicly available GWAS data set consisting of three cohorts. In both approaches, single-locus analysis (logistic regression) was conducted to filter the data with a less conservative p-value than the Bonferroni threshold; this resulted in a subset of SNPs used next in multi-locus analysis (random forest (RF)). In the second approach, we took into account prior biological knowledge, and performed sample stratification and linkage disequilibrium (LD) in addition to logistic regression analysis to preselect loci to input into the RF classifier construction step.

**Results:**

The first approach gave 199 SNPs mostly associated with genes in calcium signaling, cell adhesion, endocytosis, immune response, and synaptic function. These SNPs together with *APOE and GAB2 *SNPs formed a predictive subset for LOAD status with an average error of 9.8% using 10-fold cross validation (CV) in RF modeling. Nineteen variants in LD with *ST5, TRPC1, ATG10, ANO3, NDUFA12, and NISCH *respectively, genes linked directly or indirectly with neurobiology, were identified with the second approach. These variants were part of a model that included *APOE *and *GAB2 *SNPs to predict LOAD risk which produced a 10-fold CV average error of 17.5% in the classification modeling.

**Conclusions:**

With the two proposed approaches, we identified a large subset of SNPs in genes mostly clustered around specific pathways/functions and a smaller set of SNPs, within or in proximity to five genes not previously reported, that may be relevant for the prediction/understanding of AD.

## Background

It is predicted the number of people who suffer from Alzheimer's disease (AD) will increase from 5 million to 13.4 million in the United States of America and will be 115.4 million worldwide by 2050 [1,2]. There is currently no treatment to stop or reverse the progress of this disease. This neurodegenerative disorder is believed to be caused by an inability to clear β-amyloid (increasing all its forms: monomer, oligomer, insoluble fibrils, and plaques) from the Central Nervous System provoking neuronal impairment and cell death, and by tangled tau formation when cells are dying [3]. Genetic variation is an important contributor to the risk for this disease, estimated to be up to seventy-nine percent in the late-onset AD (LOAD) more frequent form of the disease [4]. A few genes have been confirmed by independent studies to be implicated with LOAD, summarized below.

Alzheimer's can be divided into early-onset AD (EOAD) and LOAD. There are thus far three established genes involved in EOAD and follow autosomal dominant inheritance *APP *(β-amyloid precursor protein), *PSEN1 *and *PSEN2 *(presenilin-dependent γ-secretase activity cuts amyloid precursor proteins into β-amyloid peptides) [5,6]. Another well established genetic risk factor is *APOE *(it encodes a lipoprotein that may interact with accumulated β-amyloid); it manifests in the more common LOAD and its inheritance does not follow Mendelian principles [7,8]. *APOE *has three common alleles, ε4, ε3, and ε2, and each of these variants of the gene are determined by two single nucleotide polymorphisms (SNPs). In European populations, ε4ε4 homozygotes are the most likely to develop disease, followed by ε3ε4 heterozygotes and ε3ε3 homozygotes, with ε2 heterozygotes having the least risk [8,9]. However, a person who has one or two copies of ε4 may never develop AD, while another who does not carry the ε4 alleles may [8].

*APOE *genotypes could be useful in combination with other genetic variations to predict disease risk since the scientific literature suggests the existence of additional genetic factors associated with LOAD. In the past two years, at least eight genes mapped to the immune system, cholesterol metabolism, and cell membrane processes have been confirmed by independent genome-wide association studies (GWAS) to be implicated with LOAD (See AlzGene database [10]). The genetic factors are *CLU *(it encodes apoliprotein J and may have a similar function as to that of *APOE*), *PICALM *(it encodes a protein involved in intracellular traffic of neurotransmitters between proteins and lipids), *CR1 *(it encodes the main receptor of complement C3b protein thought to be involved in β-amyloid clearance through phagocytosis) [5,11,12], *BIN1 *(it is involved in synaptic vesicle endocytocis) [5,13,14]; moreover, recently two separate studies conducted by Hollingworth P., *et al *and Naj, A.C. *et al *identified *MS4A6A/MS4A4E *(these encode cell membrane proteins), *CD2AP *(encodes a protein involved in endocytocis), *EPHA1 *(it produces a membrane bound protein involved in cell and axon guidance and synaptic function; additionally, it is involved in cell morphology, motility, and inflammation), and provided further support for *CD33 *(it is involved in cell-cell interaction and function regulation of cells in the immune system and also mediates endocytocis through a process independent from clathrin) [14,15]. Different SNPs in *CD33 *were previously identified by Bertram, L. *et al *[14-16]. *CLU, PICALM, CR1 *and *BIN1 *were confirmed by Naj, A.C. *et al *and *BIN1 *and *CR1 *were confirmed by Hollingworth P., *et al *as LOAD susceptibility loci [14,15]. In the study by Naj, A.C. *et al*, the genetic effect for the most salient SNPs at each locus had estimated population attributable fractions (PAF) of 2.72% - 5.97%; nonetheless, the authors caution that the true PAF might be different [15]. These newly confirmed genes could be mapped to pathways related to the innate and adaptive immune response - (*CLU, CR1, CD33, EPHA1*) [14,17], cell membrane processes including endocytocis (*PICALM, BIN1, CD33, CD2AP*) [14], and cholesterol/lipid metabolism (*CLU*) [14,17].

A few years ago, another gene that was shown to have an increased associated risk with LOAD was *GAB2 *although with inconsistent reproducibility by independent GWAS [18,19]. *GAB2 *protein may be involved in protection from the formation of insoluble tau deposits known as neurofibrillary tangles (NFTs) [9] and may participate in the production of β-amyloid [20]. Reiman, Eric. M. *et al *utilized stratification and linkage disequilibrium (LD) analysis and found six SNPs, part of a common haplotype block covering the *GAB2 *gene, to have a strong interaction with *APOE *in three groups of *APOE *ε4 carriers [9].

*APOE *by itself, or in combination with *GAB2*, remains to some extend a weak predictor for the risk of developing AD [8]. We used a published GWAS data set from Reiman and colleagues [9] to analyze it for AD risk determination in new loci by different models in *APOE *ε4 positive and negative samples.

One of the challenges trying to identify multiple interacting genetic mutations associated with disease in studying the etiology of complex diseases arises from the fact that there are millions of genome-wide variants, many of them untyped in the study samples of GWAS, and the number of possible combinations encountered in "interaction analysis" grows exponentially with the number of variants. As a result, it is computationally prohibitive to perform a comprehensive test for interaction analysis between four or more factors and disease. Heuristic approaches must be developed to analyze these data, that leverage and combined statistical and data mining methods [21].

We devised two informatics approaches to identify new genetic biomarkers. The first approach utilizes statistical and data mining methods. The second approach also leverages prior biological knowledge to refine the analysis. In both approaches, multi-locus (classifier building) analysis is done with a reduced number of variants that first passed, for instance, a single-locus (logistic regression) threshold.

## Results and discussion

### Approach I: Choice of SNPs without prior biological knowledge for model building

#### Step 1

In order to cast a wide net to filter the data and take into account the correlation among some of the SNPs due to LD, the association analysis was run with a p-value = 1E-3. SNPs from *APOE *and *GAB2 *were excluded from the analysis, since these are already known to be associated with AD in this data set [9]. This gave 199 SNPs with p-values ≤ 1E-3 and 1 < ORs < 5. Table 1 lists the top seven scoring SNPs; the complete list of all 199 SNPs is found in additional file 1, table SA.

**Table 1:** Logistic regression top scoring SNPs, approach I

Gene Symbol dbSNP RS ID	Distance to Gene	Unadj. p-value	FDR_BH p-value	OR (95% CI)	Pathway/Disease/function
***NISCH ***rs6784615	intron	7.16E-07	4.47E-02	2.21 (1.61-3.02)	Interaction with PAK4 for reduction of LIMK1 phosphorylation; neuronal migration and axon/dendrite outgrowth [22,23].

***RABEP1 ***rs4356530	upstream 27742	8.59E-07	4.47E-02	2.21 (1.61-3.02)	Endocytosis [24,32].

***THEMIS ***rs9398855	intron	2.25E-06	8.78E-02	2.13 (1.56-2.92)	Immnune response [25-27].

***NDUFA12*** rs249153	downstream 40719	4.13E-06	1.29E-01	1.62 (1.32-2.00)	AD, Parkinson's, Hungtinton's, oxidative phosphorylation [28-32].

***MUC21 ***rs2517509	downstream 72544	4.93E-06	1.39E-01	3.14 (1.92-5.12)	Prevention of cell-cell interaction of Integrins [55].

***TUSC1 ***rs10115381	upstream 328744	5.33E-06	1.39E-01	2.08 (1.52-2.85)	Shwachman-Diamond syndrome [56].

***CTNNA3 ***rs10996618	downstream 187123	1.19E-05	2.03E-01	2.03 (1.48-2.78)	AD (4 studies), Adherens junction, Inmune response [10,32].

FDR_BH = Benjamini & Hochberg (1995) step-up False Discovery Rate control.

#### Step 2

**Figure 1:**
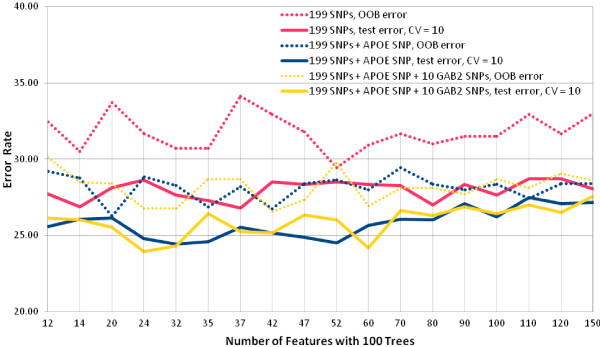
**RF performance assessment, different number of features and number of trees fixed at 100; approach I**.

**Figure 2:**
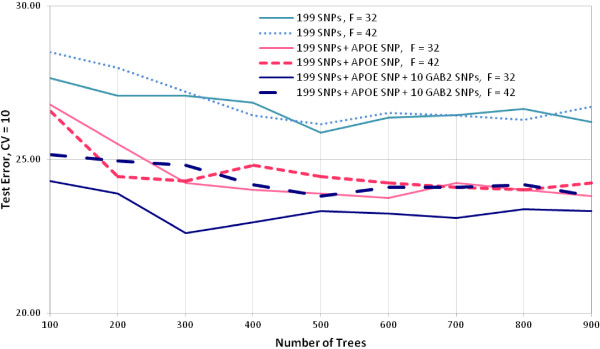
**RF tuning, best number of attributes at different number of trees; approach I**. F = number of features.

After univariate association analysis, the Random Forest (RF) classifier performance assessment was done with the 199 SNPs data. With 100 trees, Figure 1 shows the test and out of bag (OOB) errors for different number of features (SNPs). The figure suggests that increasing the number of attributes above 70 actually leads to a gradual increase in test error rate, 10-fold cross validation (CV), for the 199 SNPs and *APOE *SNP and the 199 SNPs, *APOE *SNP and *GAB2 *SNPs sets. OOB error rate (estimated class ŷ*_i _*is determined from models where row *i *is out-of- bag) is not a good estimation of test error in all instances here; however, as features are added to the forest the OOB error becomes a better estimator of the test error for these two data sets. Figure 2 shows the classifier tuning; the additional induced randomness on the selection of number of attributes for choosing the splits seems to have worked, giving a modest improvement with average 10-fold CV error rates in the range of 23-27%.

In order to further improve the classifiers, a supervised instance resample filter was applied to the data. The original case-control distribution in the data is 61% cases and 39% controls. After the data is filtered the distribution of the data becomes 52% cases and 48% controls. A big reduction in misclassification was obtained by first resampling the data to make its distribution more balanced followed by RF. The results at 100, 300, and 600 trees and various numbers of attributes are listed in Table 2. When *APOE *and *GAB2 *SNPs alone are used, the average classification error rate is 33.3%. This error rate is reduced when the 199 SNPs are used for classification. Random forests built with eighteen SNPs from the 199 SNPs give an average 10-fold CV error of 11.7%, and when the 199 SNPs are added to the bag containing either an *APOE *SNP or an *APOE *SNP and *GAB2 *SNPs the average 10-fold CV error is reduced to between 9.3% and 9.5% for 11 and 18 attributes respectively.

**Table 2:** RF modeling, filtered data, approach I

Data	RF	10-Fold	CV	%		Error
**(*APOE *ε4+ & ε4- samples)**	**Number of Trees**	**F = 7**	**F = 11**	**F = 18**	**F = 32**	**F = 42**

***APOE *& 10 *GAB2 *SNPs**						

	100	33.3	33.2			

	300	33.3	33.2			

	600	33.4	33.5			

**199 SNPs**						

	100		11.9	12.3	12.5	12.3

	300		11.3	11.3	12.1	11.6

	600		10.6	11.6	11.2	11.1

***APOE *& 199 SNPs**						

	100		10.9	9.9	10.4	10.3

	300		8.9	9.6	9.5	10.3

	600		9.0	9.1	10.1	9.9

***APOE*, 10 *GAB2*, & 199 SNPs**						

	100		9.9	10.1	9.9	11.2

	300		8.9	9.1	9.5	10.3

	600		9.1	9.4	9.9	10.3

F = number of features.

A manual compilation of pathway, disease association, or biological function information reveals some of the 199 SNPs are associated with genes involved in calcium signalling, cell adhesion, endocytosis, and immune response in addition to synaptic function. This information was added to both Table 1 and the additional file 1, table SA. Some of the genes linked to the 199 SNPs appear among genes previously identified in GWAS as posted in the AlzGene database [10]. Furthermore, some of the top 199 SNPs are novel SNPs part of or in proximity (may be acting as flags) to genes that may be engaged in a cascade of events leading to AD. Some of these genes and their relevance to AD are discussed below.

For instance, *NISCH *codes for a cytosolic protein Nischarin which negatively affects cell migration by forming inhibitory complexes with PAK family kinases among other proteins [22]. *PAK4 *suppression decreases the phosphorylation of LIMK1, key for axon/dendrite outgrowth and neuronal migration [23]. Another gene, *RABEP1 *codes for rabaptin-1 which interacts with Gap-43. One of the main roles of Gap-43 is adjustment of neurotransmitter release, endocytosis, and long-term potentiation and its expression and function is altered in AD [24]. Recently identified *THEMIS *produces a protein also known as *GAB2 *associated protein (Gasp) which plays a crucial regulatory role in positive selection during thymocyte development [25-27]. Post-positive selection, thymocytes differentiate into CD4 or CD8 single-positive (SP) thymocytes as determined by their restriction to MHC class II and I respectively. SP CD4 and CD8 in time leave the thymus for other organs and form part of the adaptive immune system. It is thought Gasp may function through a new molecular pathway downstream of T-cell receptor (TCR) signalling [27]. Further studies will be needed to establish the role this gene may play in the etiology of AD.

Changes of the expression of mitochondrial genes such as *NDUFA12*, part of complex I, may alter the oxidative metabolism in AD [28,29]. Complex I initiates electron transfer by oxidizing NADH and transferring the electrons to coenzyme Q while pumping protons across the mitochondrial membrane creating an electro-chemical proton gradient [29]. The high rate of oxygen consumption needed for normal function, polyunsaturated fatty acid and transition metal ion composition, and limited antioxidant defence mechanisms renders neurons vulnerable to oxidative damage [29,30]. Energy decline and mitochondrial dysfunction are a major, early event in AD. Complex I deficiency decreases energy production by oxidative phosphorylation this in turn increases reactive oxygen species (ROS) which often causes structural and functional cell membrane changes setting off a vicious cycle that ends in apoptosis.

Research by Rhein *et al *found tau induces mitochondrial dysfunction and increases levels of ROS and together with β-amyloid synergistically alters complex I function and energy balance with aging in AD [31]. Tau has specific sensitivity of complex I oxidative phosphorylation system. Furthermore, β-amyloid directly interacts with mitochondria via the translocase of the outer membrane (TOM) system. Additionally, maternal family history of AD links maternal inheritance of mitochondria to predisposition to AD and glucose hypometabolism [31]. *NDUFA12 *is already listed as part of the AD pathway [32].

### Approach II: Choice of SNPs with prior biological knowledge for model building

#### Step 1

Table 3 highlights 19 SNPs with the smallest p-values from the logistic regression and stratified analysis, in strong LD, and within six genes potentially relevant to AD. The LD patterns and gene overlays for the SNPs are shown in the supplementary information (additional file 2, figure S1, additional file 3, figure S2, additional file 4, figure S3, additional file 5, figure S4, additional file 6, figure S5, and additional file 7, figure S6). 

**Table 3:** CMH top scoring SNPs in LD, approach II.

Gene (Chr.) dbSNP RS ID	Physical Position	Distance to Gene	Minor Allele (MAF)	p-value from χ2	OR (95% CI)
		***APOE *ε4- SAMPLES**			

***ST5 ***(11)					

rs4910068	8830651	intron	C (0.25)	9.47E-05	1.57 (1.25-1.98)

rs10743089	8744744	intron	A (0.33)	2.12E-05	1.59 (1.28-1.97)

		***APOE *ε4+ SAMPLES**			

***TRPC1 ***(3)					

rs4259003	144006245	intron	A (0.21)	6.82E-05	2.36 (1.53-3.64)

rs9784320	144024724	intron	C (0.25)	9.68E-04	1.87 (1.28-2.71)

rs2033912	143999057	intron	T (0.22)	1.71E-03	1.86 (1.26-2.75)

rs891159	81526843	intron	C (0.24)	2.40E-05	2.34 (1.56-3.49)

***ATG10 ***(5)					

rs1485587	81362798	intron	G (0.48)	8.11E-05	1.82 (1.35-2.45)

rs4703879	81589571	intron	A (0.24)	1.01E-03	1.86 (1.28-2.71)

***ANO3 ***(11)					

rs1389421	25747721	upstream 561825	G (0.45)	3.39E-06	2.07 (1.52-2.83)

rs10834774	25715397	upstream 594149	C (0.20)	4.01E-03	1.86 (1.21-2.85)

rs11028909	25729021	upstream 580525	G (0.20)	4.13E-03	1.84 (1.21-2.81)

***NDUFA12 ***(12)					

rs249153	93848520	downstream 40719	C (0.19)	3.19E-06	1.63 (1.33-2.00)

rs249154	93848687	downstream 40552	C (0.18)	3.22E-05	1.54 (1.25-1.89)

		***APOE *ε4+ and ε4- SAMPLES**			

***NISCH ***(3)					

rs6784615	52481466	intron	C (0.09)	6.63E-07	2.13 (1.57-2.88)

rs9855470	52468315	intron	A (0.06)	4.86E-05	2.11 (1.46-3.05)

rs6445486	52481531	intron	A (0.06)	2.77E-04	1.90 (1.34-2.71)

rs10865972	52466487	intron	C (0.06)	3.38E-04	1.87 (1.32-2.65)

rs4687619	52493826	intron	T (0.06)	3.54E-04	1.93 (1.34-2.79)

rs6810027	52499614	intron	C (0.05)	5.80E-04	1.89 (1.31-2.73)

All p-values are uncorrected.

Four genes, distinct from the ones already identified in approach I, are discussed next. *ST5*, suppression of tumorigenicity 5, encodes three proteins. One of the proteins, p126, is an activator of mitogen-activated protein kinase MAPK1 also known as ERK2 [33]. ERK1 and ERK2 are some of several proline-directed kinases that have been shown to phosphorylate tau protein [34]. Tau binds and stabilizes microtubules in cells and in neurons; intracellular transport occurs in axons through microtubules [35]. Hyperphosphorylation reduces tau binding to microtubules and may increase neurofibrillary tangles (NFTs) in cell bodies and dendrites of neurons [36]. There is direct correlation between NFTs and memory decline in AD patients [36]; however, it remains to be seen how important of a role ERK2 plays in the hyperphosphorylation of tau. Another gene, *TRPC1 *codes for a TRP cation channel protein expressed in the neurons of the hippocampus and cortex among other regions of the brain. *TRPC1 *is activated by either G receptor proteins or intracellular Ca^2+ ^depletion [37]. Strübing *et al *discovered that TRPC1 channels uniquely adjust neuronal function independently of synapse processes [37]. In addition, they demonstrated that TRPC1 can form heteromeric channels i.e. TRPC1/TRPC5. TRPC5 is expressed in the hippocampus; TRPC1/TRPC5 is activated by G_q_-coupled receptors and not by Ca^2+ ^depletion and its regulation is not neurotransmitter specific [37]. Calcium signalling necessary for axonal regeneration in the adult CNS and for growth cone response of spinal neurons in *Xenopus *to myelin-associated glycoprotein is mediated by TRPC1 channels [38]. Calcium disequilibrium has been observed to lead to neuronal injury and apoptosis [39]. Key modulators of calcium homeostasis such as presenilins and CALHM1 have been associated with EOAD [39]; however, there have not been prior studies on *TRPC1 *in AD patients.

*ATG10 *is an E2-like ligase protein involved in two ubiquitin-like modifications essential for autophagosome formation [40]. Autophagy is an intracellular degradation mechanism responsible for clearance of misfolded proteins, pathogens, and organelles (organelles such as functionally disabled mitochondria in aging) [41]. Double-membrane autophagosomes enclose cytoplasmic proteins and later degrade them by fusing with lysosomes. Autophagy initiation enhances the clearance of tau and offers a cytoprotective role. Overactive or dysfunctional autophagy may promote neuronal cell death in disease states contributing to the pathology of multiple neurodegenerative disorders [42]. Blocking autophagosome formation by knockout of either *ATG5 *or *ATG7 *genes causes ubiquitinated protein aggregates and eventual neurodegeneration, demonstrating that autophagy is both constitutive and essential for neuronal functioning [42].

A genome-wide screen study by Lipinski *et al *showed that ROS are common mediators upstream of the activation of the type III PI3 kinase (critical protein in autophagy initiation) in response to β-amyloid peptide. On the other hand, lysosomal blockage also caused by β-amyloid is independent of ROS. Furthermore, they proved that autophagy is transcriptionally down-regulated during normal aging in the human brain in contrast to the autophagy up-regulation observed in later stages of AD human brains. In addition, AD drugs they tested have inhibitory effects on autophagy, decreasing input into the lysosomal system; they hypothesized this may ameliorate cellular stress in AD [43].

A fourth gene, *ANO3 *encodes anoctamin 3, rs1389421 is in a 49 kb LD region upstream of *ANO3 *as seen in additional file 5, figure S4. The anoctamin family of ten highly hydrophobic membrane proteins is also known as *TMEM16 *[44]. Some anoctamins function as Ca^2+ ^activated Cl^- ^channels (CaCCs) in the retinal photoreceptor synaptic terminals and the olfactory sensory neurons; others participate in tumor progression [44,45]. Some studies indicate that olfactory neurogenesis disruption is linked to AD [46]. *ANO2*, *3*, and *4 *are mostly expressed in neuronal tissues. *ANO3 *and *ANO4 *mRNA are equally expressed in spinal cord, brain stem, cerebellum, and eye; however, it is not known how ano3 and ano4 function thus far [44,47]. *Ist2p*, ANO in S. cerevisiae, is translated locally at the peripheral endoplasmic reticulum (ER) and may be inserted into the plasma membrane by the fusion of peripheral ER with the plasma membrane. If these anion selective proteins in mammals are transported by a similar novel mechanism, it is believed they might have effects in protein synthesis in axons and dendrites [45].

#### Step 2

The 19 SNPs in LD with six genes that resulted from the analysis in step 1 above were used next in RF. Table 4 shows that when combining the 19 SNPs with the *APOE *SNP the average 10-fold CV error rate is reduced to 20.5%. The 10-fold CV error rate is reduced to 16.9% when the data set is the 19 SNPs, *APOE *SNP, and 10 *GAB2 *SNPs, and using 600 trees and 11 features for tree building. The higher 10-fold CV error rate obtained in approach II as compared to that of approach I may not be due to LD. As Meng, Y.A. *et al *explain, in RF if a SNP is near the root of a tree in the forest and a second SNP in LD with the first SNP is close to the leaf of the same tree, the permutation of the first SNP value will not increment the prediction error of the tree because the second SNP can be a substitute for the first SNP. However, the prediction error might be still somewhat increased [48].

**Table 4:** RF modeling, filtered data, approach II.

Data	RF	10-Fold	Cross	Validation	% Error
**(*APOE *ε4+ and ε4- samples)**	**Number of Trees**	**F = 6**	**F = 7**	**F = 11**	**F = 18**

***APOE *& 10 *GAB2 *SNPs**					

	100		33.3	33.2	

	150		33.4	33.5	

	200		33.4	33.2	

	250		33.2	33.2	

	300		33.3	33.2	

	600		33.4	33.5	

**19 SNPs**					

	100	26.9		27.5	27.9

	150	26.7		27.0	27.5

	200	26.8		27.0	27.4

	250	27.1		27.3	27.6

	300	27.2		27.1	27.6

	600	27.1		27.4	27.4

***APOE *& 19 SNPs**					

	100	20.3		20.8	20.7

	150	19.9		20.6	20.8

	200	20.1		20.7	20.9

	250	20.3		20.6	20.6

	300	20.1		20.6	20.6

	600	19.8		20.4	20.7

***APOE*, 10 *GAB2*, & 19 SNPs**					

	100	17.6		17.6	18.2

	150	17.2		17.0	17.6

	200	17.2		17.2	18.1

	250	17.3		17.1	17.9

	300	17.2		17.3	17.9

	600	17.3		16.9	17.6

F = number of features

## Conclusions

It is believed that LOAD is a complex disease caused by the interaction of multiple genetic and environmental factors. In the past two years, at least eight genes have been confirmed to be associated with LOAD. These are common risk variants of moderate to small effects the same as *APOE*. The new variants functionality could be mapped to the immune response, cholesterol metabolism, and cell membrane processes pathways [14]. However, a percentage of AD heritability is still missing. The purpose of this study was to explore new associations between multiple SNPs and AD by data mining approaches. We analyzed a published AD GWAS data set by a couple of two-step approaches that first filtered the data with a low threshold to obtain a data subset used in a second step for multi-locus analysis. In one approach statistical and data mining techniques were implemented, and in the other approach biological domain knowledge and LD analysis were done prior to the multi-locus analysis. A 10-fold CV was done with the multi-locus analysis which helped remove bias from the reported error rate. Previously AD associated SNPs [7,9] were removed from the data to avoid obscuring other possible significant variants. There is overlap between the SNPs identified with both approaches; some of the genes associated with the SNPs used to build the classifiers have not been reported before as currently listed in the AlzGene database [10].

The model built for approach I confirmed, that *APOE *and *GAB2 *genotypes alone can produce a moderate determinant of LOAD status [9], being able to discriminate between cases and controls with about 33% error rate (10 fold CV). By adding close to 200 other genome-wide SNPs that had a relatively high score of association with LOAD from the GWAS data set, the error rate of the model was greatly reduced, from 33% to about 10%. While many of the 200 SNPs were in the vicinity of genes that could potentially be involved in AD pathways, some of them were not.

The model built for approach II leveraged biological domain knowledge to select a small number of SNPs from genes that had relevance to LOAD. This model used only 19 to 30 SNPs (Table 4), while the model in approach I used one order of magnitude more, about 200 SNPs (Table 2). The model in approach II was less successful in lowering the LOAD classification error rate - to only about 17%, vs. the 10% that the model in approach I did. Approach II; however, with its limited number of biologically-relevant SNPs, would be much easier to test, as opposed to the model in approach I, which included 200 genome-wide SNPs. A model employing dozens of SNPs might be harder to test, but it could be that dozens of genetic variants linked to many different pathways could be involved in the etiology of AD as is the case for other complex diseases [19], and as it is beginning to emerge from the GWAS outcomes from the past two years.

In order to improve the results from the analysis of this data, a (joint) meta-analysis could be done with another AD data set conducted on the same platform. The combined data sets would give more statistical power for gene-gene interaction effects and make possible fine mapping of variants with larger effect sizes. The functionality of the selected SNPs here could be further assessed by mapping the variants to genes that interact with or are in the same pathways as those already implicated in AD, and querying of genomic annotations of SNPs representing variation in microRNA target sites.

The two approaches described here are only a starting point that can be further refined to better understand the possible causes of LOAD. Similar approaches - that combine high throughput genomics techniques, statistical and data mining analysis, and leverage biological domain knowledge - can be applied to study other complex diseases that have a strong genetic component.

## Methods

### Data

A published AD GWAS data set was obtained from the Translational Genomics Research Institute [9]. The data includes results on 312,316 SNPs that passed quality control checks across the genome genotyped with the Mapping 500K Array set from Affymetrix on 1411 LOAD cases and controls from a discovery group and two replicate groups. Each of the three groups is divided into two sub-groups of *APOE *ε4 carriers and ε4 non-carriers. In addition to genotypes, the original data includes phenotypes such as gender, age of disease onset, and age at death. The analysis presented here focuses on genotype interactions and excludes these phenotypes from the analysis.

The data can be described as 312,316 nominal predictors along with a two class response variable y. The response variable is unevenly distributed; it is 61% cases and 39% controls. Also, the data has missing values. Furthermore, in order to avoid false-positive results due to population stratification, the data is from a Caucasian population of European ancestry; the samples were obtained from the United States and from the Netherlands.

The data was originally used to identify a novel interaction between LOAD and two genes, *APOE *and *GAB2 *[9]. In this analysis, known *GAB2 *and *APOE *SNPs are first excluded and then re-added in the model building phase. In the next section, we describe the approaches employed and briefly explain the RF algorithm.

### Analytical approaches for Alzheimer's disease association analysis

In order to identify new genetic variants that increase disease risk, we implement some of the latest algorithm versions for disease association, LD, and data mining with the most recent genetic variant annotation files. A two step analysis, to reduce multiple genetic interactions to be tested, is implemented by two approaches: one statistically driven and a second incorporating sample stratification and biological knowledge. In the first step of both approaches, SNPs are filtered at a less stringent threshold for disease association. The multiple testing threshold correction, Bonferroni, assumes there are M independent tests (α_p _= α_e _/M where α_p _is the point-wise error and α_e _is the experimental error); however, the independence assumption fails in genetic association studies since there is correlation among some of the SNPs due to LD. Thus, we use a threshold of p-value = 1E-3 and take into account positive ORs (a positive OR means the minor allele increases disease risk relative to the major allele) for the genome-wide screening step. Furthermore, for the first step of approach II, the data is also filtered by the p-values and ORs from chi-square tests, and by significant LD values of selected SNPs within or close (~5 Kb) to neurobiological relevant genes. For both two step methods, known *GAB2 *and *APOE *SNPs (originally published with this data set [9]) are first removed so they do not obscure the finding of other statistically significant SNPs and then they are re-added in the RF building phase.

Random forest (or random forests) is an ensemble classifier that consists of many decision trees and outputs the class that is the mode (most frequent outcome) of the class's output by individual trees [49]. Ensemble methods use multiple models to obtain better predictive performance than could be obtained from any of the constituent models. For example, if individual classifiers would have an error rate of ε = 0.35, an ensemble of twenty-five independent base classifiers will make a wrong prediction at a smaller rate of 0.06 by the formula $\sum_{i=13}^{25}\left(\begin{array}{c} 25\\ i \end{array}\right)\varepsilon^{i}{\left(1-\varepsilon \right)}^{25-i}=0.06$[50].

RF is a special case of bagging algorithm which is simple to train and tune. Bagging, or bootstrap aggregating, is a parallel ensemble method that induces additional randomness by allowing bag size to be chosen [51]. For each classifier in the ensemble, a sample is drawn uniformly and with replacement from the original training data set. If the training data has more rows expressing cases than expressing controls, the randomness causes more frequent cases rows in the bag than control rows. This results in cases rows getting classified much better than the control rows. The aim is to have both classes classified in a way to lead to overall low error rate. RF, as a classifier, induces additional randomness in the selection of features from a subset for deciding the splits at the nodes in each tree and if the skewed features in the data are de-selected one may improve the model predictions or vice versa. The size of the subset is decided by first taking the square root of the total number of attributes in the data set or by the log_2 _of the total number of attributes + 1. The additional randomness in RF helps to reduce variance (correlation among attributes) and maintain bias. It is standard to let the trees grow deep and not to prune them since the average of trees that are put together is taken to reduce the variance.

In the second step for both approaches, RF algorithms are optimized to build stable classifiers with new SNPs, the most significant *APOE *SNP (rs4420638) identified by Coon *et al *[7], and 10 *GAB2 *SNPs (Table 1[9]) for AD prognosis.

### Approach I: Choice of SNPs without prior biological knowledge for model building

#### Step 1

Logistic regression with a less stringent p-value than the Bonferroni cut-off of 0.05/312,316 = 1.55E-7 is performed using PLINK v 1.07 [52] on all samples. A filter is set at a p-value = 1E-3 and the association analysis is run with removal of *APOE *and *GAB2 *SNPs. In addition, SNPs with p-values ≤ 1E-3 and 1 < ORs < 5 are selected. The raw genotype data corresponding to each of the selected SNPs is extracted by running Perl scripts and PLINK code. And to identify the genes corresponding to these SNPs, annotation files updated by the chip manufacturer (Affymetrix) with the Human Genome v 19 are queried.

#### Step 2

After pre-formatting the data subset, the data mining analysis is run using WEKA v 3-6-6 [53]. All the analysis in this study involves a cross validation of 10 folds without pruning the trees. To start the RF model building, the number of SNPs to include in the model is calculated by taking the square root of the total number of SNPs. Four data sets are used to build the RF classifiers, and all of the sets have y (disease) in the last column as class attribute. The first data set includes the *APOE *SNP and 10 *GAB2 *SNPs, the second set consists of the SNPs from step 1, the SNPs from step 1 plus the *APOE *SNP form the third set, and the fourth set comprises the SNPs from step 1, the *APOE *SNP, and 10 *GAB2 *SNPs. In order to assess the performance of each of the classifiers, the number of trees is held constant at 100 and the number of features (SNPs) is varied. Then, the classifiers are tuned by holding constant the various numbers of attributes, which gave the smallest test and OOB error rates (for the three data sets) in the performance step, and by changing the number of trees.

A supervised instance resample filter is applied to each data set. This produces a random subsample of each data set using sampling with replacement. The filter is set to bias the class distribution towards a uniform distribution; the original case-control distribution in the data is 61% cases and 39% controls.

After classification modelling, a manual compilation of epistasis information relevant to AD on the 199 SNPs is done.

### Approach II: Choice of SNPs with prior biological knowledge for model building

#### Step 1

For the second approach, prior biological knowledge is used to supplement statistical analysis in selecting SNPs from genes that are more likely to play a role in AD. SNPs from *APOE *and *GAB2 *are excluded; then, the data is filtered by p-values ≤ 1E-3 and 1 < ORs < 5 from logistic regression and the Cochran-Mantel-Haenszel (CMH) test. For CMH, the stratification (three groups) is done based on *APOE *ε4 carrier status using PLINK.

A list of twenty-three SNPs selected from logistic regression and CMH based on their p-values and ORs, and situated in the vicinity of genes potentially relevant to AD is uploaded into Haploview v 4.2 [54] in order to find their linkage to other SNPs within a 300 kb region. The default settings are kept; the "Download HapMap info Track", release 22 version 2 and release 21 with panel CEU (Caucasian European), and the "Solid Spine" method to detect strong LD are utilized [54].

#### Step 2

The RF is implemented at various fixed number of attributes and trees with four different data sets. The *APOE *SNP and 10 *GAB2 *SNPs are a first set, the SNPs in LD from step 1 are a second set, and the SNPs from step 1 together with the *APOE *SNP are a third set. The SNPs from step 1 added to the *APOE *SNP and *GAB2 *SNPs make a fourth set. The classification building is performed in the same manner as for approach I - step 2.

## Competing interests

The authors declare that they have no competing interests.

## Authors' contributions

Conceived and designed the experiments: VD. Analyzed the data and wrote the paper: NB. Made major edits: VD. Both authors read and approved the manuscript.

## Pre-publication history

The pre-publication history for this paper can be accessed here:

http://www.biomedcentral.com/1471-2350/13/7/prepub

## Supplementary Material

Additional file 1 **Table SA**. Complete list of 199 SNPs from logistic regression approach I with corresponding pathway, disease or biological function information which may be pertinent to AD. Click here for file

Additional file 2 **Figure S1**. LD display for SNPs across the 300 kb region surrounding the *ST5 *locus. Top: Entrez gene track overlaid with Hapmap genotyped SNPs across a 300 kb pairs interval surrounding the *ST5 *locus. Bottom: zoomed LD SNP region. The SNPs identified, rs4910068 and rs10743089, were found to be in significant LD with a D' value of 0.83. Standard color scheme for Haploview: D' < 1 and LOD < 2 are white, D' = 1 and LOD < 2 are blue, D' < 1 and LOD ≥ 2 are shades of pink/red, D' = 1 and LOD ≥ 2 are bright red. LOD = log of the odds. Click here for file

Additional file 3 **Figure S2**. LD display for SNPs across the 300 kb region surrounding the *TRPC1 *locus. Top: Entrez gene track overlaid with Hapmap genotyped SNPs across a 300 kb region surrounding the *TRPC1 *locus. Bottom two: zoomed LD SNP region. The SNPs identified, rs4259003, rs9784320, and rs2033912, were found to be in significant LD; rs4259003 and rs9784320, rs4259003 and rs2033912, and rs9784320 and rs2033912 with D' values of 1.0. Standard color scheme for Haploview: D' < 1 and LOD < 2 are white, D' = 1 and LOD < 2 are blue, D' < 1 and LOD ≥ 2 are shades of pink/red, D' = 1 and LOD ≥ 2 are bright red. LOD = log of the odds. Click here for file

Additional file 4 **Figure S3**. LD display for SNPs across the 300 kb region surrounding the *ATG10 *locus. Top: Entrez gene track overlaid with Hapmap genotyped SNPs across a 300 kb region surrounding the *ATG10 *locus. Bottom three: zoomed LD SNP region. The SNPs identified, rs891159, rs1485587, and rs4703879, were found to be in significant LD; rs891159 and rs1485587, rs891159 and rs4703879, and rs1485587 and rs4703879 with D' = 1. Standard color scheme for Haploview: D' < 1 and LOD < 2 are white, D' = 1 and LOD < 2 are blue, D' < 1 and LOD ≥ 2 are shades of pink/red, D' = 1 and LOD ≥ 2 are bright red. LOD = log of the odds. Click here for file

Additional file 5 **Figure S4**. LD display for SNPs across the 300 kb region surrounding the *ANO3 *locus. Top: Entrez gene track overlaid with Hapmap genotyped SNPs across a 300 kb region surrounding the *ANO3 *locus. Bottom two: zoomed LD SNP region. The SNPs identified, rs1389421, rs10834774, and rs11028909, were found to be in significant LD; rs1389421 and rs10834774, rs1389421 and rs10834774, and rs11028909 and rs10834774 with D' = 1. Standard color scheme for Haploview: D' < 1 and LOD < 2 are white, D' = 1 and LOD < 2 are blue, D' < 1 and LOD ≥ 2 are shades of pink/red, D' = 1 and LOD ≥ 2 are bright red. LOD = log of the odds. Click here for file

Additional file 6 **Figure S5**. LD display for SNPs across the 300 kb region surrounding the *NDUFA12 *locus. Top: Entrez gene track overlaid with Hapmap genotyped SNPs across a 300 kb pairs interval surrounding the *NDUFA12 *locus. Bottom: zoomed LD SNP region. The SNPs identified, rs249153 and rs249154, were found to be in significant LD with D' = 1. Standard color scheme for Haploview: D' < 1 and LOD < 2 are white, D' = 1 and LOD < 2 are blue, D' < 1 and LOD ≥ 2 are shades of pink/red, D' = 1 and LOD ≥ 2 are bright red. LOD = log of the odds. Click here for file

Additional file 7 **Figure S6**. LD display for SNPs across the 300 kb region surrounding the *NISCH *locus. Top: Entrez gene track overlaid with Hapmap genotyped SNPs across a 300 kb region surrounding the *NISCH *locus. Bottom: zoomed LD SNP region. The SNPs identified, rs6784615, rs9855470, rs6445486, rs10865972, rs4687619, and rs6810027, were found to be in significant LD; rs6784615 and rs9855470, rs6784615 and rs6445486, rs6784615 and rs10865972, rs6784615 and rs4687619, rs6784615 and rs6810027, rs9855470 and rs6445486, rs9855470 and rs10865972, rs9855470 and rs4687619, rs9855470 and rs6810027, rs6445486 and rs10865972, rs6445486 and rs4687619, rs6445486 and rs6810027, rs10865972 and rs4687619, rs10865972 and rs6810027, and rs4687619 and rs6810027 with D' values of 1. Standard color scheme for Haploview: D' < 1 and LOD < 2 are white, D' = 1 and LOD < 2 are blue, D' < 1 and LOD ≥ 2 are shades of pink/red, D' = 1 and LOD ≥ 2 are bright red. LOD = log of the odds. Click here for file
