# Supplementary figures and images for: Data mining of high density genomic variant data for prediction of Alzheimer's disease risk
Source: BMC Med Genet. 2012 Jan 25;13:7. doi: 10.1186/1471-2350-13-7 (PMC3355044; doi:10.1186/1471-2350-13-7)

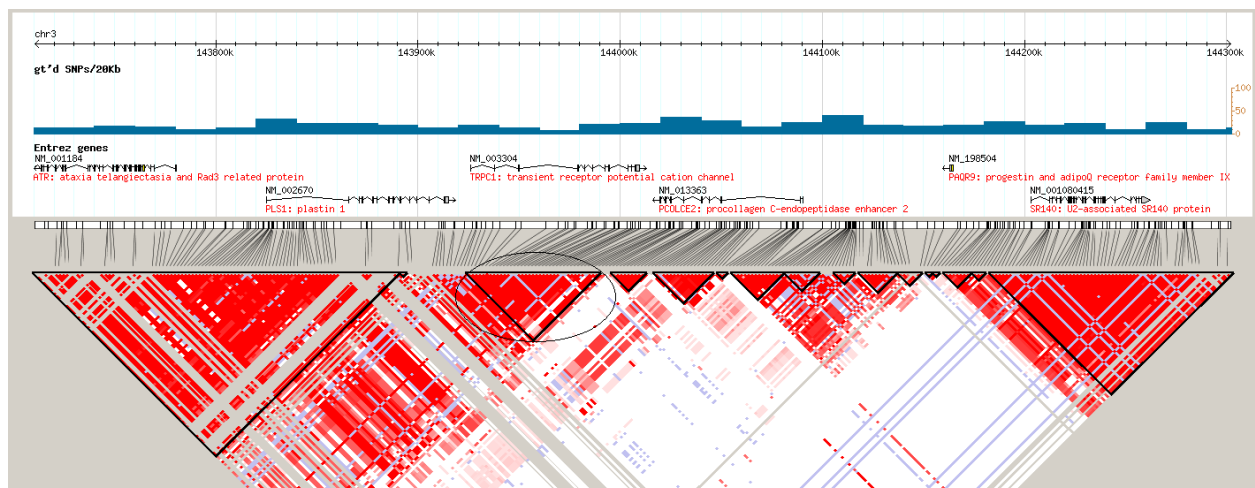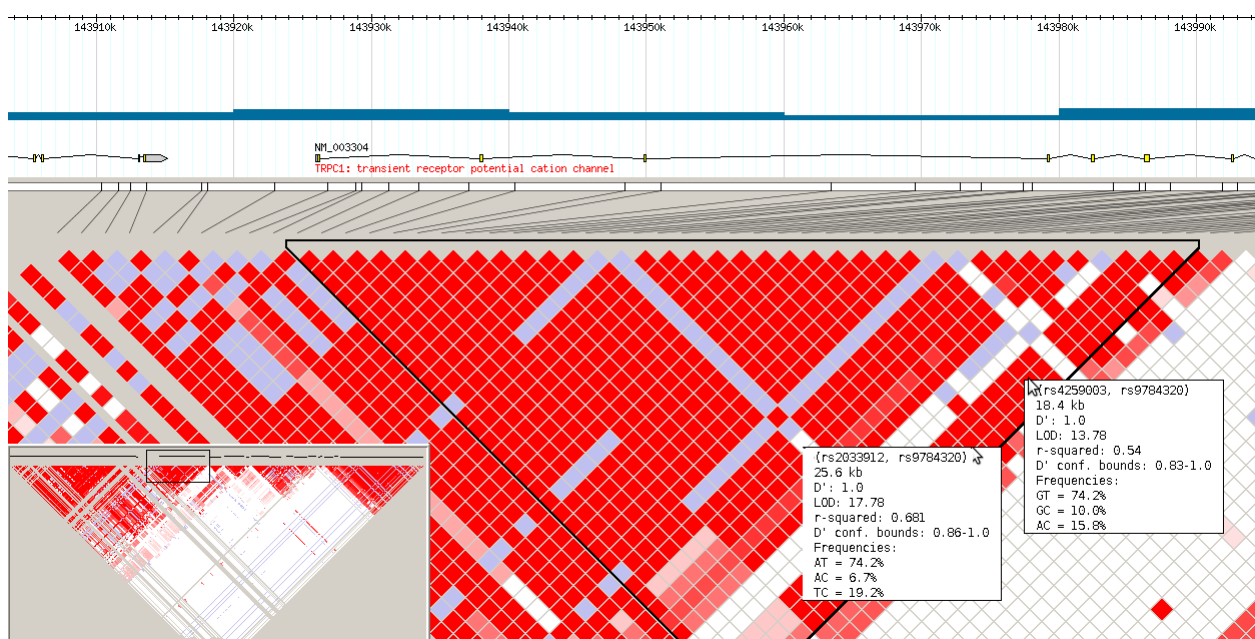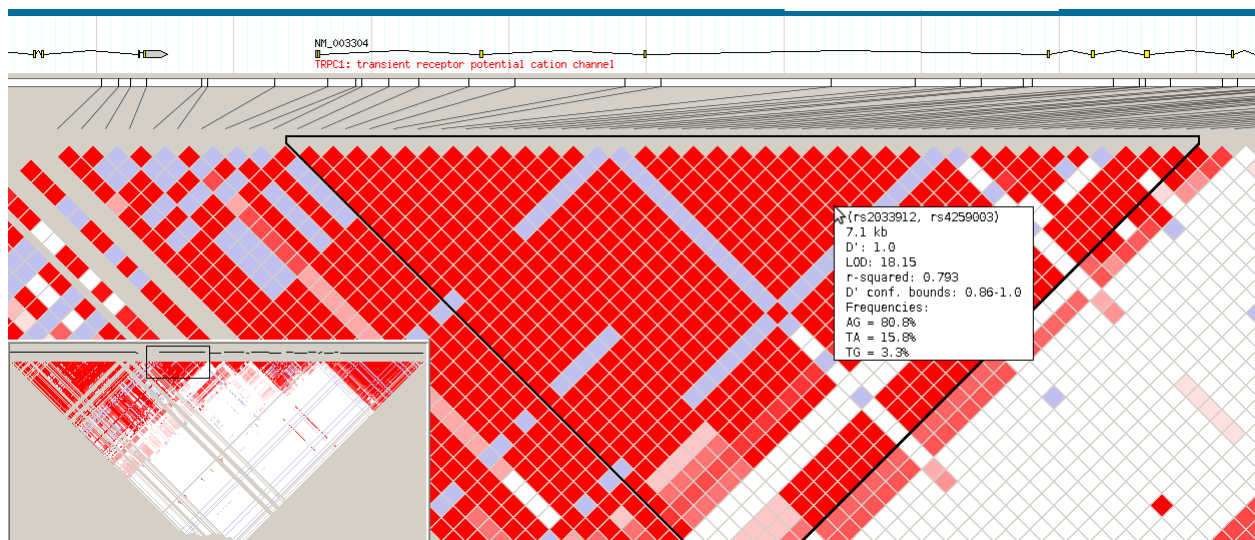

Supplement: Additional file 3 — Figure S2. LD display for SNPs across the 300 kb region surrounding the TRPC1 locus. Top: Entrez gene track overlaid with Hapmap genotyped SNPs across a 300 kb region surrounding the TRPC1 locus. Bottom two: zoomed LD SNP region. The SNPs identified, rs4259003, rs9784320, and rs2033912, were found to be in significant LD; rs4259003 and rs9784320, rs4259003 and rs2033912, and rs9784320 and rs2033912 with D' values of 1.0. Standard color scheme for Haploview: D' < 1 and LOD < 2 are white, D' = 1 and LOD < 2 are blue, D' < 1 and LOD ≥ 2 are shades of pink/red, D' = 1 and LOD ≥ 2 are bright red. LOD = log of the odds. [file 1471-2350-13-7-S3.PDF]

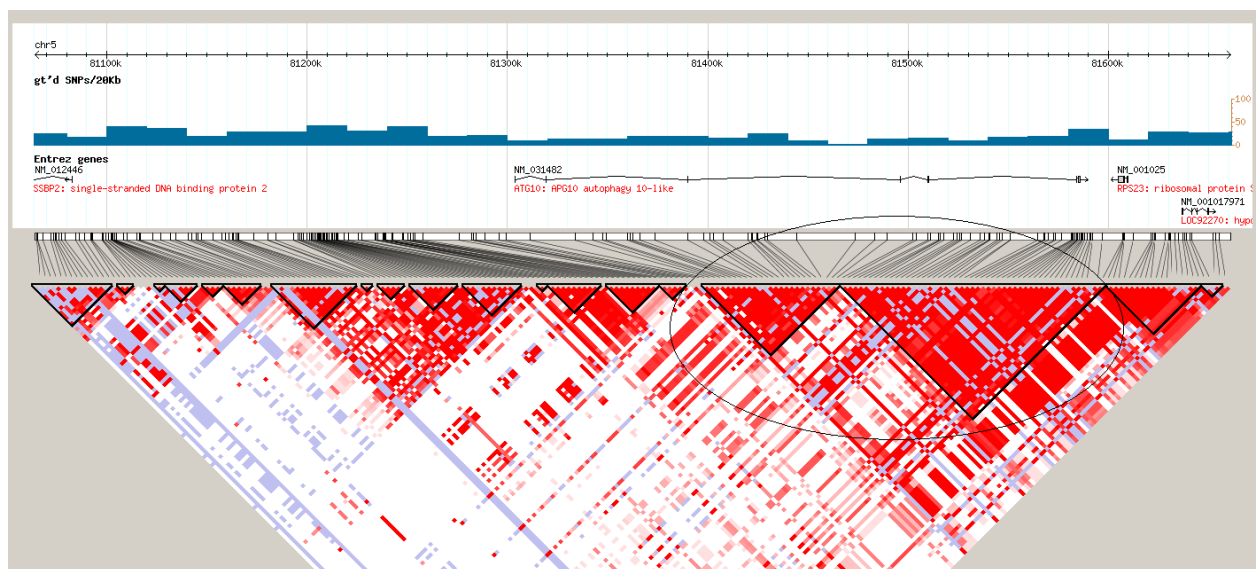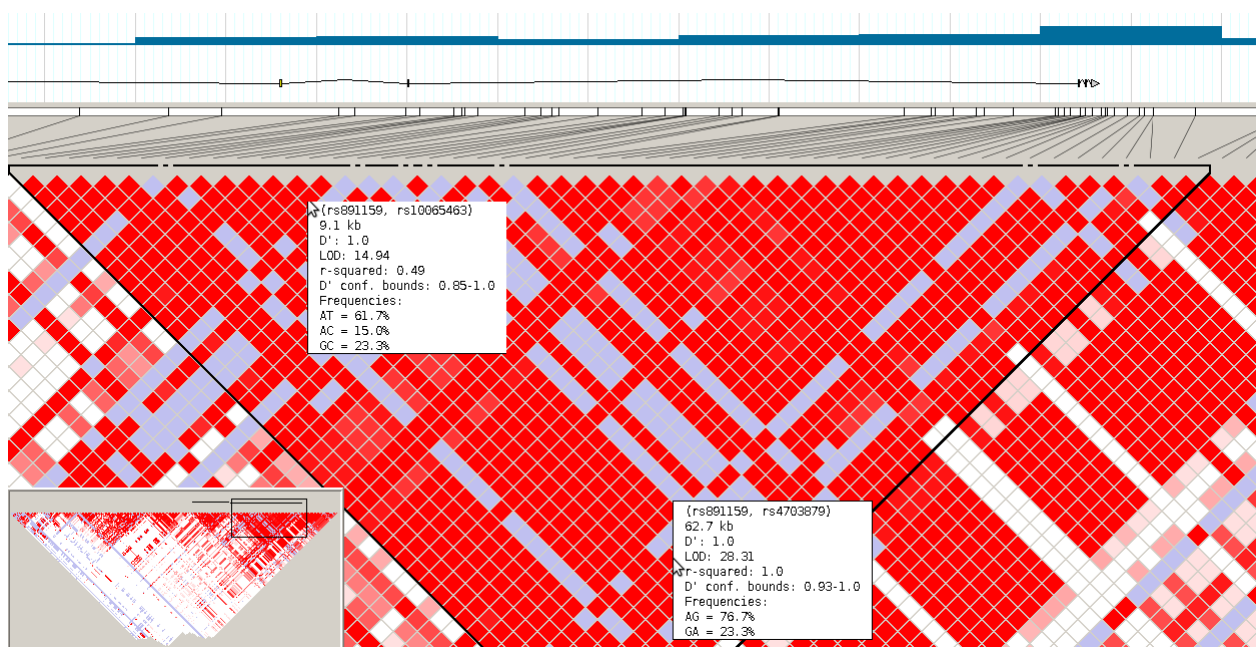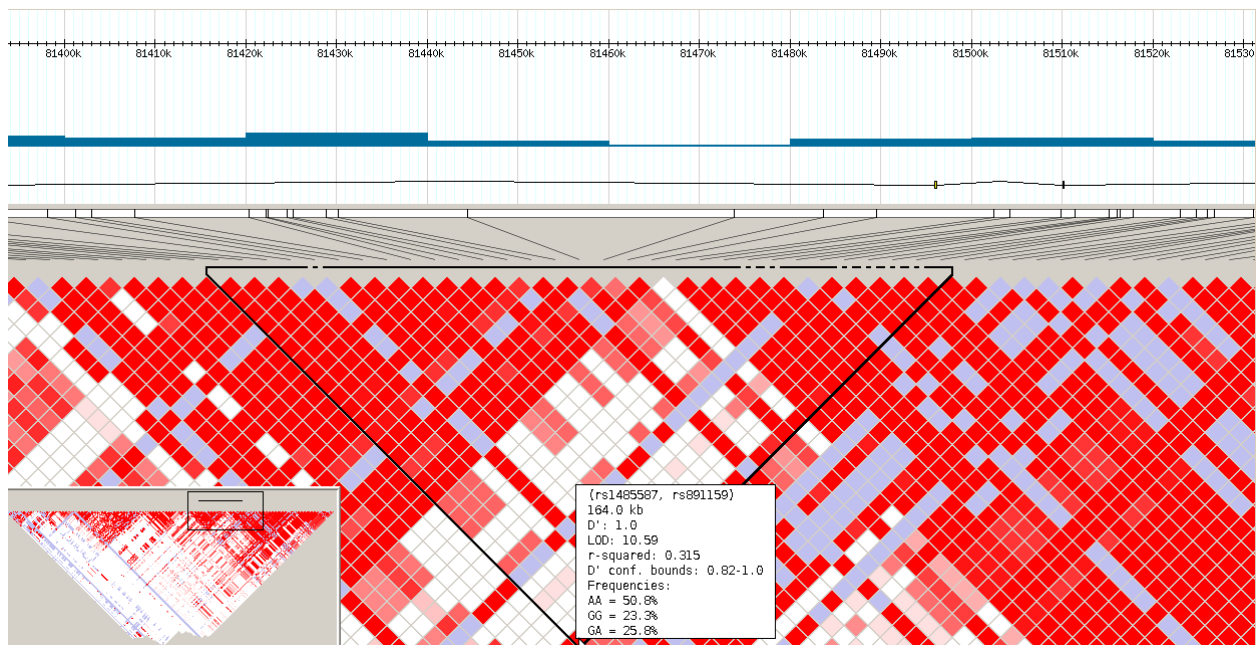

0k 81350k 81360k 81370k 81380k 81390k 81400k 81410k 81420k 81430k 81440k 81450k 81460k 81470k 81480k 81490k 81500k 81510k 81520k 81530k 81540k 81550k 81560k 81570k 8

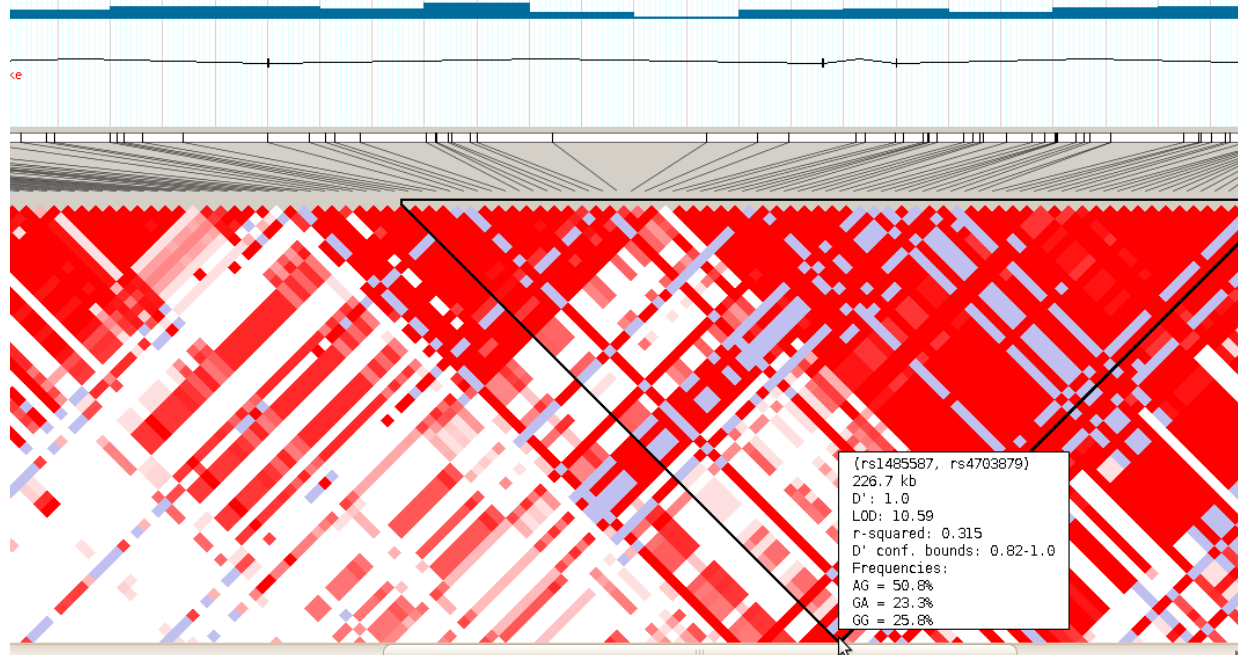

Supplement: Additional file 4 — Figure S3. LD display for SNPs across the 300 kb region surrounding the ATG10 locus. Top: Entrez gene track overlaid with Hapmap genotyped SNPs across a 300 kb region surrounding the ATG10 locus. Bottom three: zoomed LD SNP region. The SNPs identified, rs891159, rs1485587, and rs4703879, were found to be in significant LD; rs891159 and rs1485587, rs891159 and rs4703879, and rs1485587 and rs4703879 with D' = 1. Standard color scheme for Haploview: D' < 1 and LOD < 2 are white, D' = 1 and LOD < 2 are blue, D' < 1 and LOD ≥ 2 are shades of pink/red, D' = 1 and LOD ≥ 2 are bright red. LOD = log of the odds. [file 1471-2350-13-7-S4.PDF]

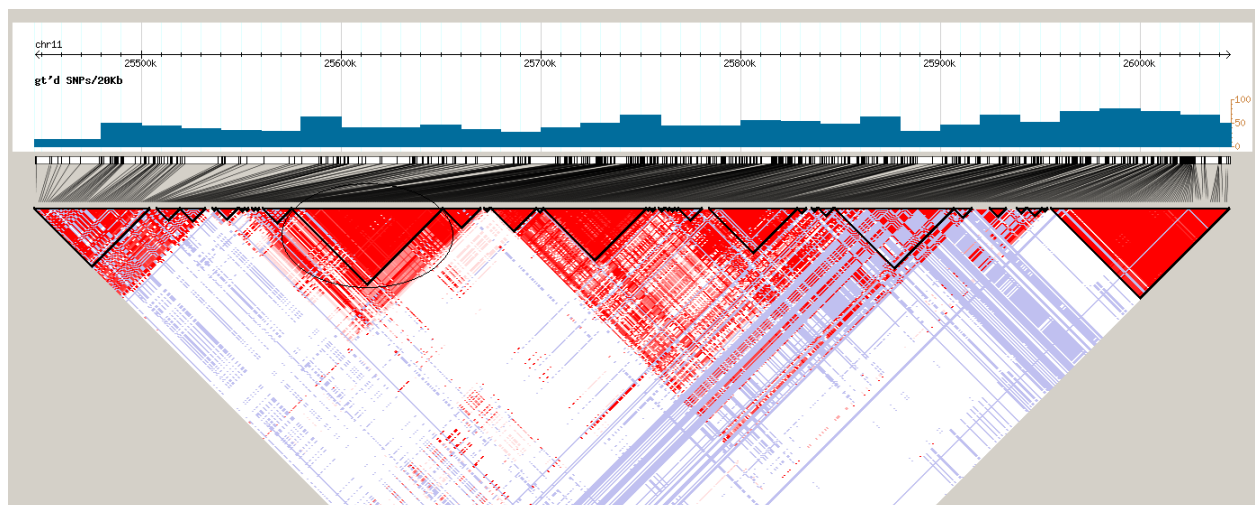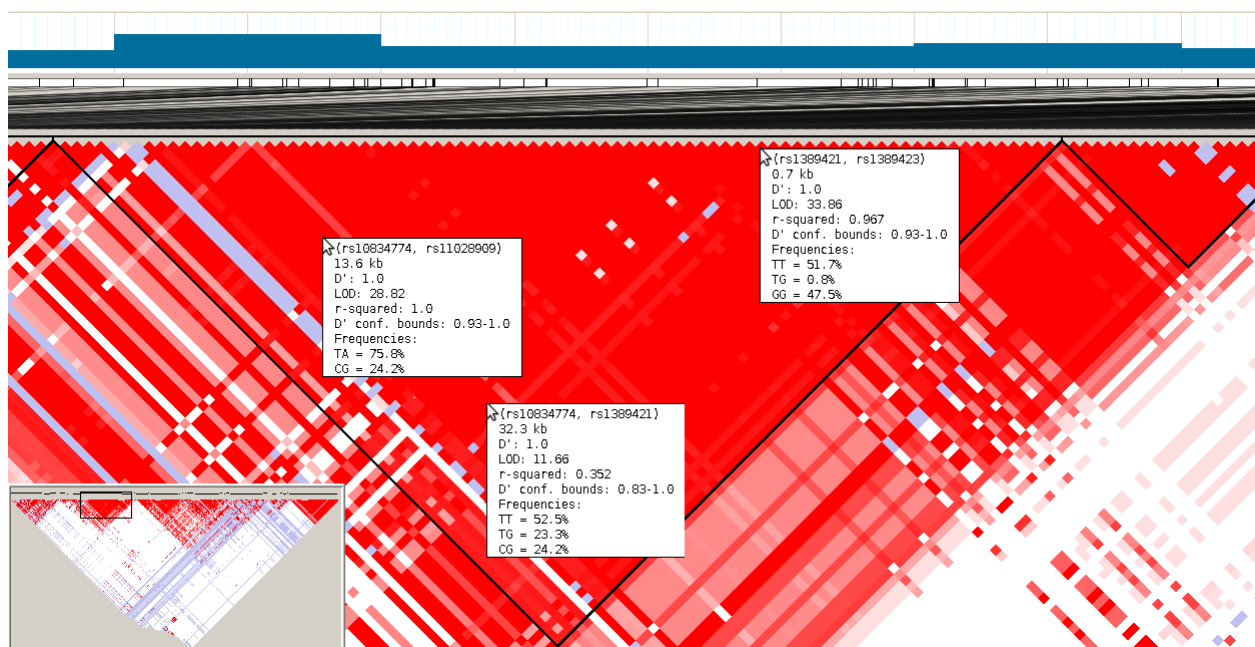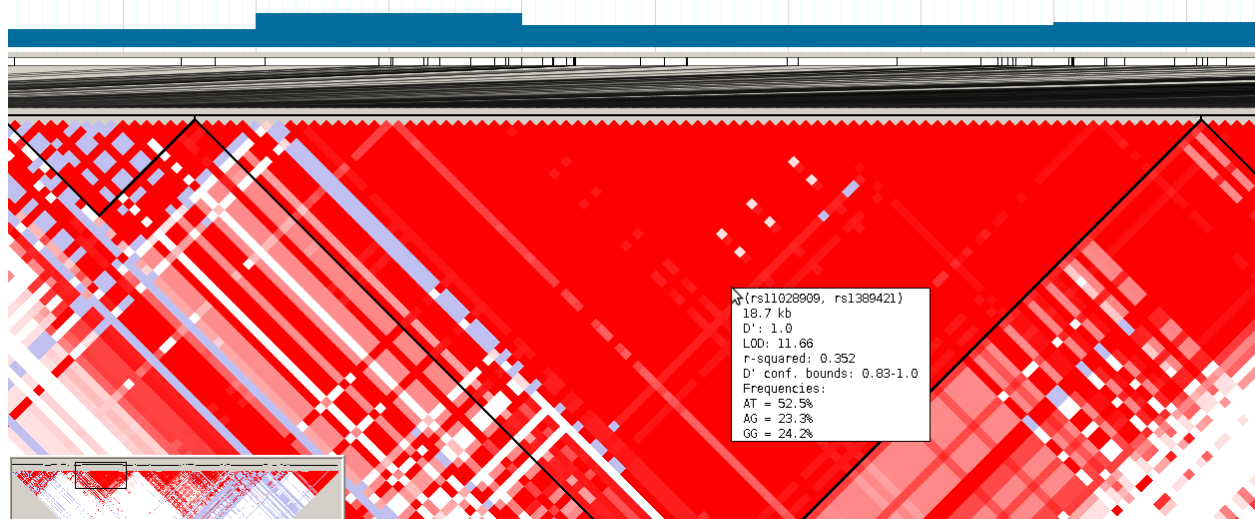

Supplement: Additional file 5 — Figure S4. LD display for SNPs across the 300 kb region surrounding the ANO3 locus. Top: Entrez gene track overlaid with Hapmap genotyped SNPs across a 300 kb region surrounding the ANO3 locus. Bottom two: zoomed LD SNP region. The SNPs identified, rs1389421, rs10834774, and rs11028909, were found to be in significant LD; rs1389421 and rs10834774, rs1389421 and rs10834774, and rs11028909 and rs10834774 with D' = 1. Standard color scheme for Haploview: D' < 1 and LOD < 2 are white, D' = 1 and LOD < 2 are blue, D' < 1 and LOD ≥ 2 are shades of pink/red, D' = 1 and LOD ≥ 2 are bright red. LOD = log of the odds. [file 1471-2350-13-7-S5.PDF]

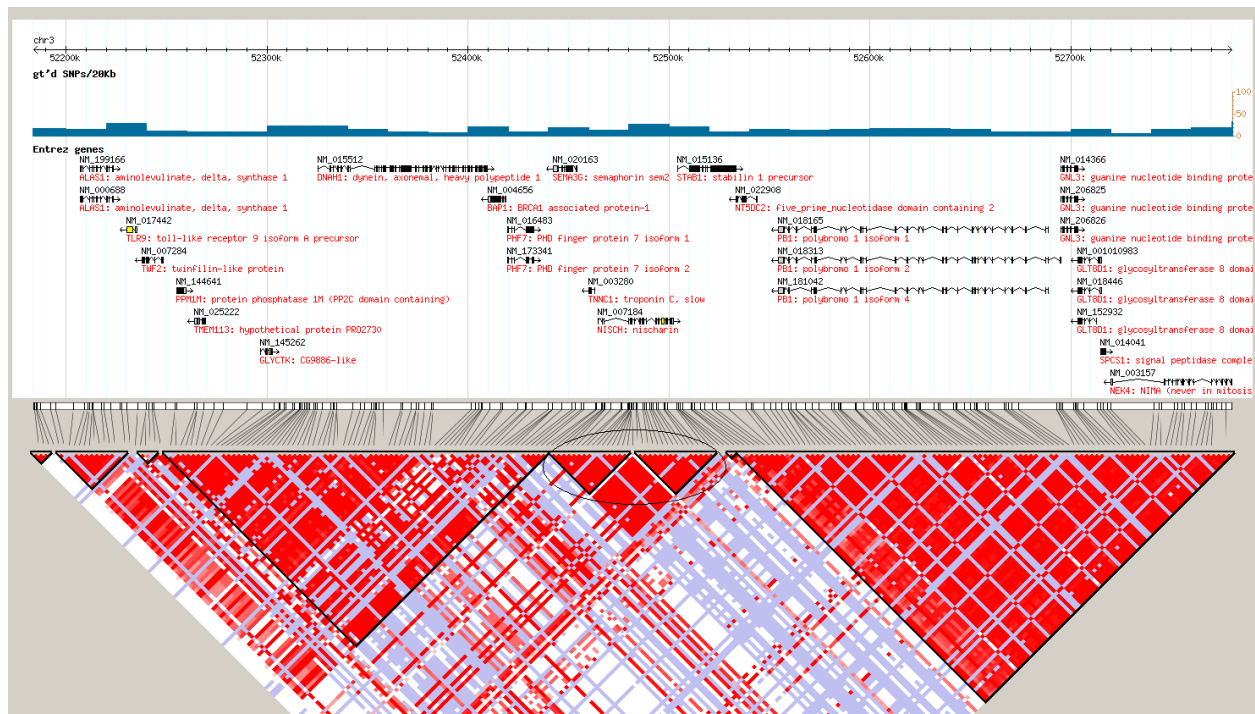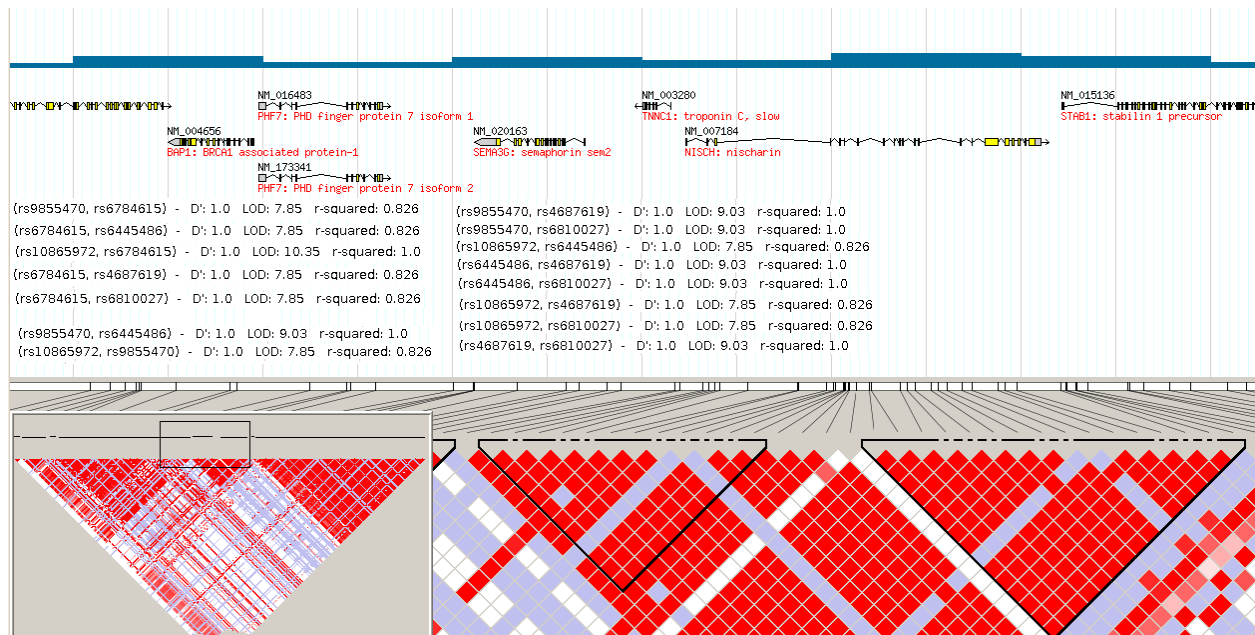

Supplement: Additional file 7 — Figure S6. LD display for SNPs across the 300 kb region surrounding the NISCH locus. Top: Entrez gene track overlaid with Hapmap genotyped SNPs across a 300 kb region surrounding the NISCH locus. Bottom: zoomed LD SNP region. The SNPs identified, rs6784615, rs9855470, rs6445486, rs10865972, rs4687619, and rs6810027, were found to be in significant LD; rs6784615 and rs9855470, rs6784615 and rs6445486, rs6784615 and rs10865972, rs6784615 and rs4687619, rs6784615 and rs6810027, rs9855470 and rs6445486, rs9855470 and rs10865972, rs9855470 and rs4687619, rs9855470 and rs6810027, rs6445486 and rs10865972, rs6445486 and rs4687619, rs6445486 and rs6810027, rs10865972 and rs4687619, rs10865972 and rs6810027, and rs4687619 and rs6810027 with D' values of 1. Standard color scheme for Haploview: D' < 1 and LOD < 2 are white, D' = 1 and LOD < 2 are blue, D' < 1 and LOD ≥ 2 are shades of pink/red, D' = 1 and LOD ≥ 2 are bright red. LOD = log of the odds. [file 1471-2350-13-7-S7.PDF]
