# Supplementary material for: Data mining of high density genomic variant data for prediction of Alzheimer's disease risk
Source: BMC Med Genet. 2012 Jan 25;13:7. doi: 10.1186/1471-2350-13-7 (PMC3355044; doi:10.1186/1471-2350-13-7)
Supplement: Additional file 2 — Figure S1. LD display for SNPs across the 300 kb region surrounding the ST5 locus. Top: Entrez gene track overlaid with Hapmap genotyped SNPs across a 300 kb pairs interval surrounding the ST5 locus. Bottom: zoomed LD SNP region. The SNPs identified, rs4910068 and rs10743089, were found to be in significant LD with a D' value of 0.83. Standard color scheme for Haploview: D' < 1 and LOD < 2 are white, D' = 1 and LOD < 2 are blue, D' < 1 and LOD ≥ 2 are shades of pink/red, D' = 1 and LOD ≥ 2 are bright red. LOD = log of the odds. [file 1471-2350-13-7-S2.PDF]

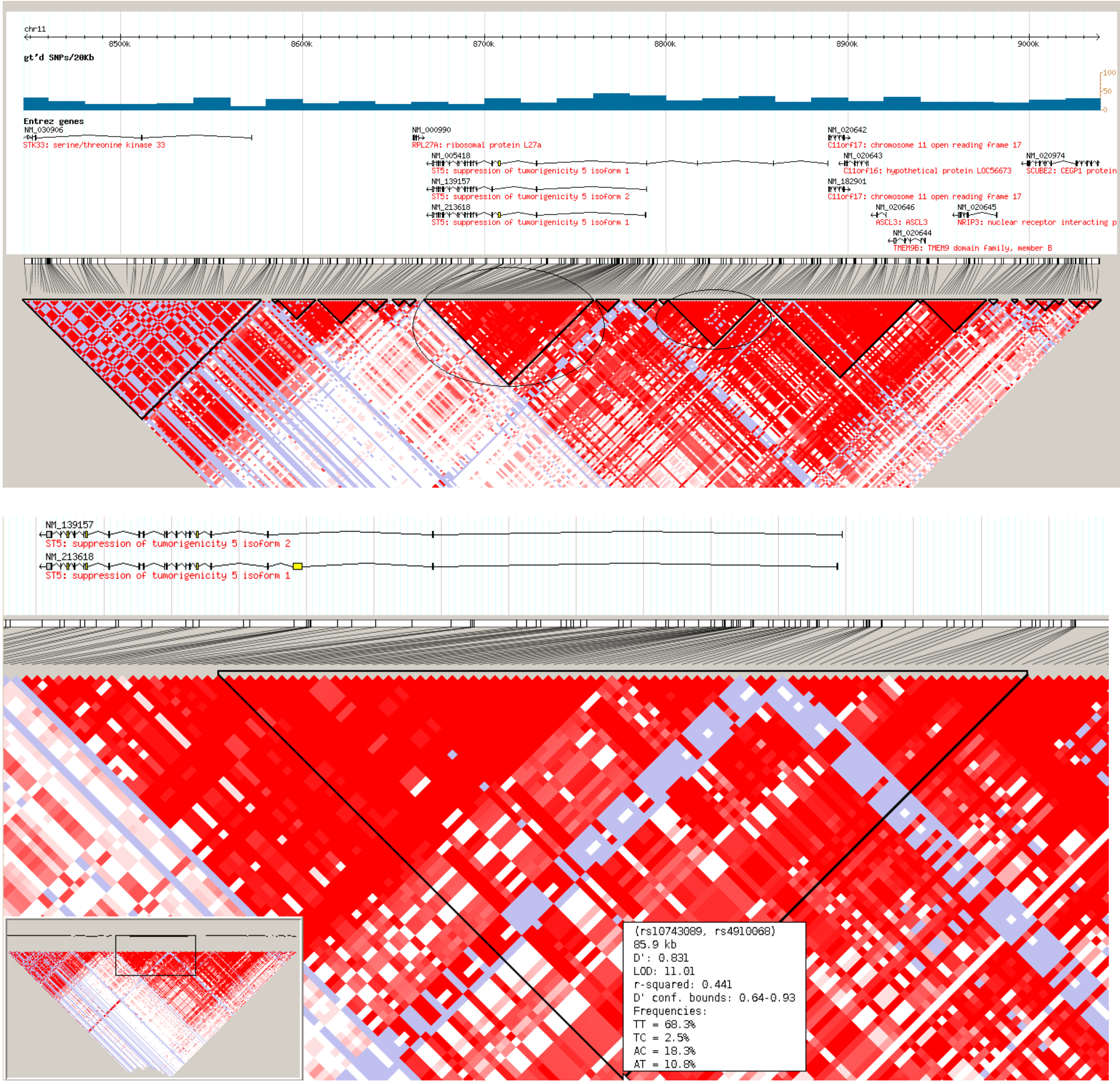

chr11
8500k
8600k
8700k
8800k
8900k
9000k
gt'd SNPs/20Kb
100
50
0
Entrez genes
NM_030906
STK33: serine/threonine kinase 33
NM_000990
RPL27A: ribosomal protein L27a
NM_005418
ST5: suppression of tumorigenicity 5 isoform 1
NM_139157
ST5: suppression of tumorigenicity 5 isoform 2
NM_213618
ST5: suppression of tumorigenicity 5 isoform 1
NM_020642
C11orf17: chromosome 11 open reading frame 17
NM_020643
C11orf16: hypothetical protein LOC56673
NM_020974
SCUBE2: CEGP1 protein
NM_182901
C11orf17: chromosome 11 open reading frame 17
NM_020646
ASCL3: ASCL3
NM_020645
NRIP3: nuclear receptor interacting p
NM_020644
TMEM9B: TMEM9 domain family, member B
NM_139157
ST5: suppression of tumorigenicity 5 isoform 2
NM_213618
ST5: suppression of tumorigenicity 5 isoform 1
(rs10743089, rs4910068)
85.9 kb
D': 0.831
LOD: 11.01
r-squared: 0.441
D' conf. bounds: 0.64-0.93
Frequencies:
TT = 68.3%
TC = 2.5%
AC = 18.3%
AT = 10.8%
