# Supplementary material for: Data mining of high density genomic variant data for prediction of Alzheimer's disease risk
Source: BMC Med Genet. 2012 Jan 25;13:7. doi: 10.1186/1471-2350-13-7 (PMC3355044; doi:10.1186/1471-2350-13-7)
Supplement: Additional file 6 — Figure S5. LD display for SNPs across the 300 kb region surrounding the NDUFA12 locus. Top: Entrez gene track overlaid with Hapmap genotyped SNPs across a 300 kb pairs interval surrounding the NDUFA12 locus. Bottom: zoomed LD SNP region. The SNPs identified, rs249153 and rs249154, were found to be in significant LD with D' = 1. Standard color scheme for Haploview: D' < 1 and LOD < 2 are white, D' = 1 and LOD < 2 are blue, D' < 1 and LOD ≥ 2 are shades of pink/red, D' = 1 and LOD ≥ 2 are bright red. LOD = log of the odds. [file 1471-2350-13-7-S6.PDF]

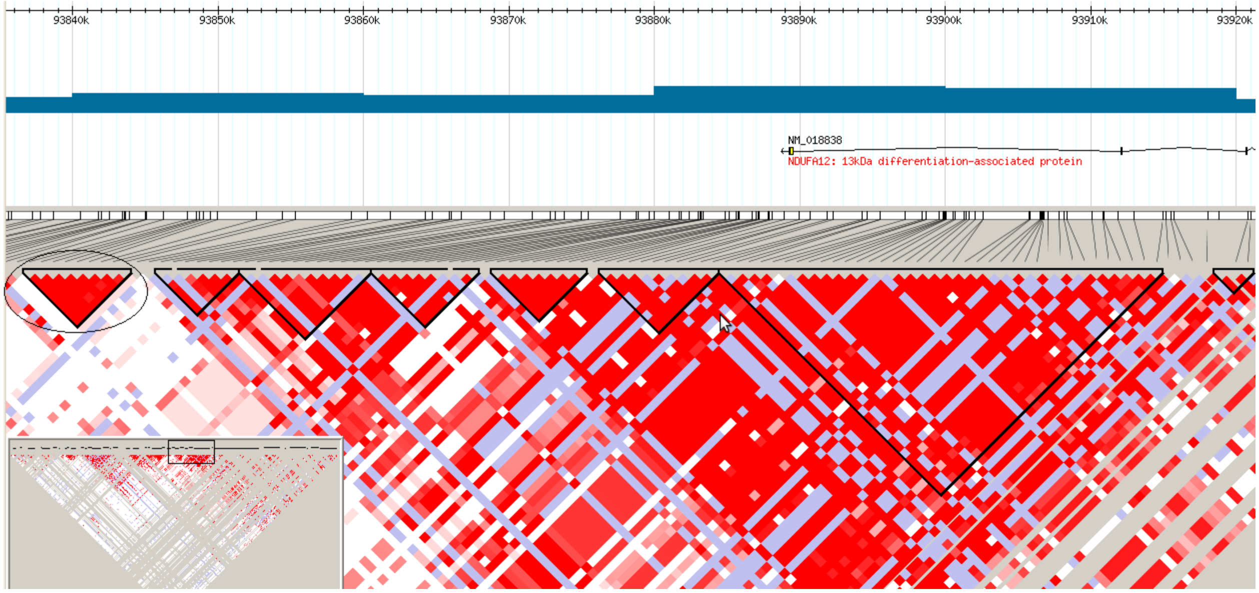

93840k
93850k
93860k
93870k
93880k
93890k
93900k
93910k
93920k
NM_018838
NDUFA12: 13kDa differentiation-associated protein

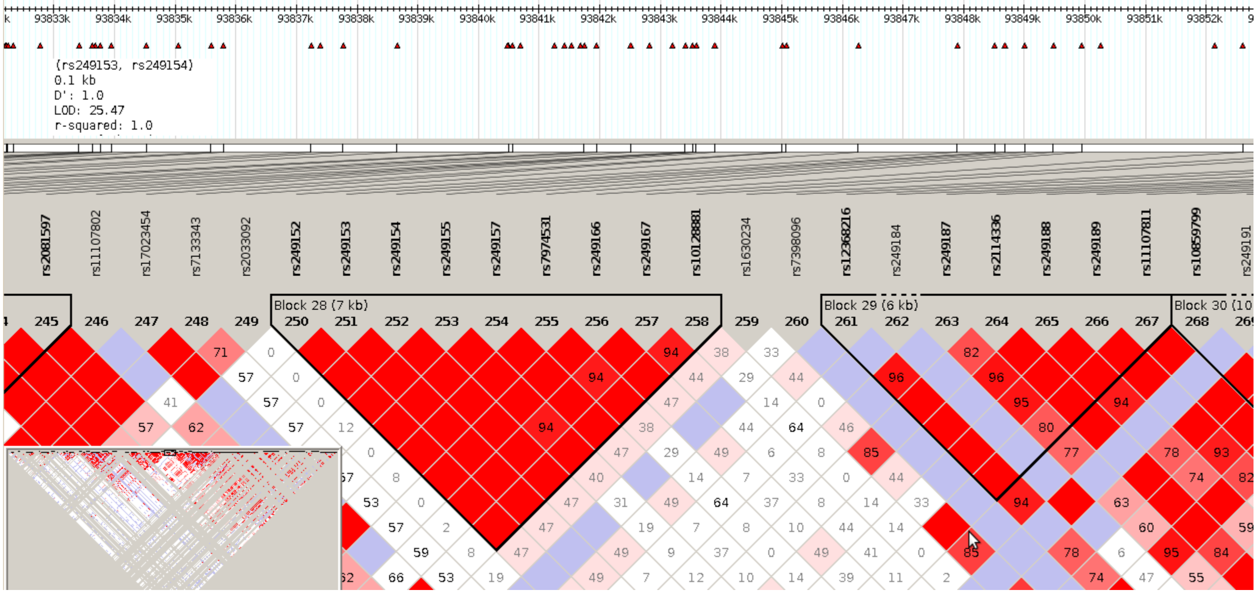

93833k
93834k
93835k
93836k
93837k
93838k
93839k
93840k
93841k
93842k
93843k
93844k
93845k
93846k
93847k
93848k
93849k
93850k
93851k
93852k
(rs249153, rs249154)
0.1 kb
D': 1.0
LOD: 25.47
r-squared: 1.0
rs2081597
rs11107802
rs17023454
rs7133343
rs2033092
rs249152
rs249153
rs249154
rs249155
rs249157
rs7974531
rs249166
rs249167
rs10128881
rs1630234
rs7398096
rs12368216
rs249184
rs249187
rs2114336
rs249188
rs249189
rs11107811
rs10859799
rs249191
Block 28 (7 kb)
Block 29 (6 kb)
